# Microgrooves and Microrugosities in Titanium Implant Surfaces: An In Vitro and In Vivo Evaluation

**DOI:** 10.3390/ma12081287

**Published:** 2019-04-19

**Authors:** Sergio Alexandre Gehrke, José Henrique Cavalcanti de Lima, Fernando Rodriguez, José Luis Calvo-Guirado, Jaime Aramburú Júnior, Leticia Pérez-Díaz, Patricia Mazón, Juan Manuel Aragoneses, Piedad N. De Aza

**Affiliations:** 1 Department of Research, Biotecnos, Cuareim 1483, Montevideo CP 11100, Uruguay; rodrigueznando@hotmail.com; 2 Department of Oral and Implant Surgery, Faculty of Health Sciences, Universidad Católica de Murcia (UCAM), 30107 Murcia, Spain; jlcalvo@ucam.edu; 3 Instituto de Bioingenieria, Universidad Miguel Hernández, Avda. Ferrocarril s/n, 03202 Elche (Alicante), Spain; pmazon@umh.es (P.M.); piedad@umh.es (P.N.D.A.); 4 Department of Rehabilitation, Universidade Federal Fluminense, Rio de Janeiro 24220-900, Brazil; josehenriquecavalcanti@yahoo.com.br; 5 Private Practice, Rivera 40004, Uruguay; 6 Department of Surgery, Faculty of Veterinary, Faculty of Itapiranga, Itapiranga 89896-000, Brazil; jaimearamburujunior@gmail.com; 7 Laboratorio de Interacciones Molecular, Facultad de Ciencias, Universidad de la Republica, Calle Iguá 4225, Montevideo 11400, Uruguay; letperez@gmail.com; 8 Department of Dental Research, Universidad Federico Henriquez y Carvajal (UFHEC), Santo Domingo 10107, Dominican Republic; jaragoneses@ufhec.edu.do

**Keywords:** dental implants, osseointegration, bone healing, surface treatment, microgrooves

## Abstract

The physical characteristics of an implant surface can determine and/or facilitate osseointegration processes. In this sense, a new implant surface with microgrooves associated with plus double acid treatment to generate roughness was evaluated and compared in vitro and in vivo with a non-treated (smooth) and double acid surface treatment. Thirty disks and thirty-six conical implants manufactured from commercially pure titanium (grade IV) were prepared for this study. Three groups were determined, as described below: Group 1 (G1), where the samples were only machined; group 2 (G2), where the samples were machined and had their surface treated to generate roughness; and test group 3 (G3), where the samples were machined with microgrooves and the surface was treated to generate the roughness. For the in vitro analysis, the samples were submitted to scanning microscopy (SEM), surface profilometry, the atomic force microscope (MFA) and the surface energy test. For the in vivo analyses, thirty-six implants were placed in the tibia of 9 New Zealand rabbits in a randomized manner, after histological and histomorphometric analysis, to determine the level of contact between the bone and implant (BIC%) and the bone area fraction occupancy (BAFO%) inside of the threads. The data collected were statistically analyzed between groups (*p* < 0.05). The in vitro evaluations showed different roughness patterns between the groups, and the G3 group had the highest values. In vivo evaluations of the BIC% showed 50.45 ± 9.57% for the G1 group, 55.32 ± 10.31% for the G2 group and 68.65 ± 9.98% for the G3 group, with significant statistical difference between the groups (*p* < 0.0001). In the BAFO% values, the G1 group presented 54.97 ± 9.56%, the G2 group 59.09 ± 10.13% and the G3 group 70.12 ± 11.07%, with statistical difference between the groups (*p* < 0.001). The results obtained in the evaluations show that the surface with microgrooves stimulates the process of osseointegration, accelerating the healing process, increasing the contact between the bone and the implant and the area of new bone formation.

## 1. Introduction

The rehabilitation of dental losses through osseointegrated implants has reached a fairly high level of confidence and has been used as a frequent treatment option in dentistry. For implantology concepts, the process of osseointegration for titanium implants is defined by the union between the implanted material and the bone tissue. The adequate union between them depends on the physical and chemical characteristics presented by the material, the surgical technique used and the patient’s conditions (general and local) [1,2,3,4]. Regarding the composition of the material, titanium is now used due to its excellent biological and mechanical properties [5,6], even when special treatments are not made on its surface (implants with smooth surfaces).

However, to improve the events required by the osseointegration process and to increase the quantity and quality of the union between the bone tissue and the surface of the implant, in view of possible adverse conditions, numerous propositions of implant surface changes were proposed [7]. Several studies have shown that physical changes (roughness) and chemical changes (adhesion of substrates) can improve and/or accelerate the osseointegration process. Among these modifications of the surface characteristics of titanium implants, this may be carried out by additive methods (titanium or hydroxyapatite deposition) [8,9] or by subtractive methods (chemical attack, blasting or laser) [7,10,11].

Some in vitro assays, such as surface microscopy, rugosity and wettability (surface energy), should be used to describe the proposed modifications and/or changes and thus be able to compare the results with other publications. Of these tests, surface wettability is the assay that may help to understand the behavior and ease (or not) of cell adhesion on the surface of the implant. Several studies have shown that there is a relationship between surface energy and cell adhesion on the surface [12,13,14].

The events related to optimal bone tissue healing using a treated implant surface are still unclear. The first stages of the osseointegration of implants involve some biological phases, such as protein adsorption, cell-surface interaction, progenitor cell recruitment and differentiation, and tissue formation at the interface between the body and the implanted material, which can be directly influenced by the physical-chemical characteristics of the material surface [15,16,17].

Although many findings on the behavior of different surfaces of titanium implants have already been made, there are still several points that need further scientific evidence, such as the relationship between cell culture results and the responses of these materials after being implanted in living organisms. In this way, based on cellular studies (in vitro), in which the benefits and the possibility of directing (guiding) the cellular growth with the elaboration of microgrooves on the surface of the implants were demonstrated [15,18,19], we proposed the present animal study to evaluate and compare in vivo the influence of a surface with the microgrooves modification plus micorugosities on the osseointegration, when compared to that of machined and treated implants without microgrooves. Previously, analyses were carried out to characterize and compare the three surfaces studied.

## 2. Materials and Methods

**Figure 1:**
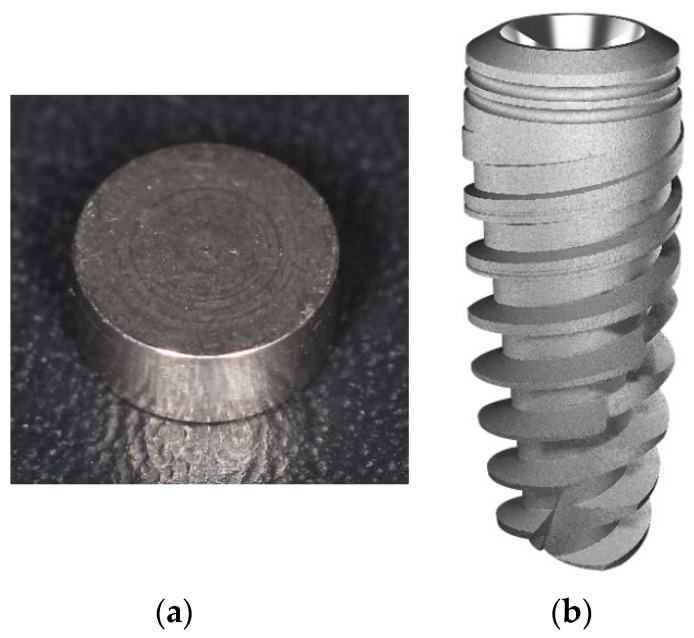
Representative image of the titanium disk (**a**) and the titanium implant; (**b**) macrodesign used for all sample groups.

Materials and groups division: Thirty disks and thirty-six conical implants, manufactured from commercially pure grade IV titanium (Derig Produtos Odontológicos Ltd.a, São Paulo, SP, Brazil) were used in this study. The prepared disks were 5 mm in diameter and 2 mm in thickness (*n* = 10 per group) and the implants were 8.5 mm in length, 3.50 mm in diameter and used a conical design (dynamic implant, Derig, São Paulo, Brazil) (*n* = 12 per group). All the implants used presented the same macrogeometric design (Figure 1), varying only the surface treatment per implant.

The samples were divided into three groups in accordance with the surface treatment applied, which are as follows: Group 1 (G1), where the samples were only machined; group 2 (G2), where the samples were machined and had their surface treated to generate roughness; and group 3 (G3), where the samples were machined with microgrooves and the surface was treated to generate the roughness. The microgrooves (lines) of the G3 group had a maximum depth of 10 µm and a 10 µm distance between them during the machining of the implants in a CNC (Computer Numerical Control)machine (Traub TNL 12, Rostock, Germany). The treatment of the surface of the G2 and G3 groups was performed with double acid conditioning, using hydrofluoric acid (HF) plus sulfuric acid (H_2_SO_4_), with controlled time and temperature, as determined by the manufacturing company (Derig, São Paulo, Brazil), as described below: The implants were immersed in an 9.0 wt.% HF solution at ambient temperature for 45 s. The second immersion was made for 30 min at ambient temperature in a 30 wt.% H_2_SO_4_ solution containing 0.09 wt.% HF. Then, all samples were treated (washed, decontaminated, sterilized and packaged) in accordance with the sanitary standards required for the commercialization of the implantable products.

**Figure 2:**
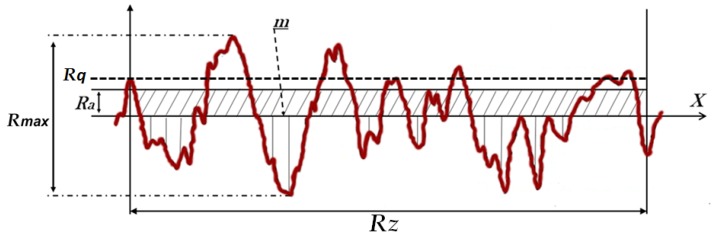
Scheme of the measured roughness parameters from the samples of each group.

Surface morphology analysis: Five titanium disks from each group were evaluated through SEM analysis (model JSM 5200, JEOL Ltd., Tokyo, Japan) to obtain images and compare the morphological characteristics of the surfaces with different increases. Five other titanium disk samples from each group were used to determinate the roughness characteristics of the surface using a series of 3D images through AFM (Atomic Force Microscopy) analysis (Agilent Technologies, AFM 5500, Chandler, AZ, USA). These same samples disks were used to measure the surface roughness parameters (Ra, Rq, Rz and Rmax) using an optical laser profilometer (Perthometer S2, Mahr GmbH, Göttingen, Germany), where Ra is the absolute value of all profile points, Rq is the root-mean-square of the values of all points, Rz is the value of the absolute heights of the five highest peaks and the depths of the five deepest valleys and Rmax is the value between the maximum valley and maximum peak. These parameters are shown in the scheme in Figure 2.

**Figure 3:**
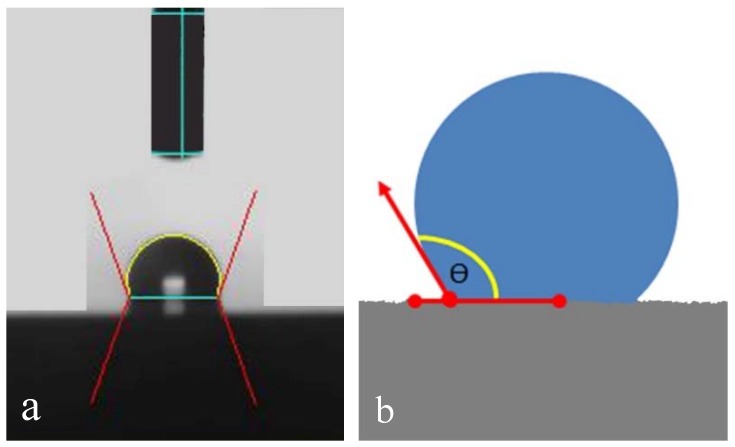
Image of the drop deposition on the surface of the disk (**a**) and a schematic image showing the measuring of the contact angle formed between the drop and the disk surface (**b**).

Surface wettability analysis: Surface wettability analysis was performed using a contact angle goniometer (Ramé-Hart Instrument Co., Succasunna, NJ, USA), which analyzes the static contact angles after the application of a drop of 5 μL of distilled water on the surface of the titanium disks, determining their hydrophilicity. Five disks from each group were used to make these measurements. The surface tension was calculated by measuring the contact angle formed between the drop and the disk surface (Figure 3). Images of the drop and the surface were taken at 0, 15, 30 and 60 s after application to analyze the stability of the drop [20]. The images were analyzed using the software DSA3 (Krüss GmbH, Hamburg, Germany).

Animal selection, surgical management and care: Nine white rabbits (New Zealand), weighing between 4 and 4.5 kg, were used for the in vivo analysis. The animals received the standard care and management applied in previous studies performed by our research group [1,2,3]. The international guidelines of animal studies were applied. The study was approved by the Animal Experimentation Committee (#004-09-2015), Faculty of Itapiranga (Itapiranga, Brazil). Thirty-six conical titanium implants (*n* = 12 per group) were installed in both tibias (*n* = 2 per tibia). The randomized distribution of the implants was performed using the site www.randomization.com. For the surgical procedures, the animals were anesthetized through the intramuscular injection of a combination of 0.35 mg/kg of ketamine (Ketamina Agener^®^; Agener União Ltd.a., São Paulo, Brazil) and 0.5 mg/kg of xylazine (Rompum^®^ Bayer S.A., São Paulo, Brazil). In both medial area of the tibias, the hairs were scraped to facilitate surgical procedures and to avoid contamination. These areas were cleaned with a povidone-iodine solution. Then, the incision was performed with an extension of ~30 mm in length in each tibia and from 10 mm of the knee position to the distal direction. The soft tissues were separated and the bone was exposed. The beds to insert the titanium implants were prepared using the drill sequence and speed, determined by the manufacturer of the implant system, under intense distilled water cooling. The implants were manually inserted with ~15 N of torque and 10 mm between them (the first implant was installed ~10 mm from the articulation). The suture was made using a simple point with nylon 4-0 (Ethicon, Johnson & Johnson Medical, New Brunswick, USA). A single postoperative dose of 0.1 mL/kg of Benzetacil (Bayer, São Paulo, Brazil) was administered intramuscularly (I/M) in each animal. For the control of pain, the animals received three I/M anti-inflammatory doses (one per day) of 3 mg/kg of ketoprofen (Ketoflex, Mundo Animal, São Paulo, Brazil). All animals were euthanized 6 weeks after the implantation surgeries using an overdose of anesthesia. Then, the bone blocks of both tibias were removed and immediately immersed in a formaldehyde solution.

Histomorphometric and histological analysis: Three days after fixation in formaldehyde solution, the samples were washed in running tap water every 12 h and then gradually dehydrated in a progressive series of ethanol solution (60% to 100%). After dehydration, the blocks (bones with the implant) were embedded in historesin (Technovit 7200 VLC, Kultzer and Co., Wehrhein, Germany), polymerized and cut in the central region of the implants using a metallographical cutter (Isomet 1000; Buehler, Germany). Then, the samples were polished using a sequence of abrasive paper (180 to 1200 mesh) in a polishing machine (Polipan-U, Panambra Zwick, São Paulo, Brazil). The samples were stained using a picrosirius hematoxylin staining technique, as described below: First, the slides were dipped through either a methanol or ethanol gradient series (100%, 90%, 80%, 70%, 60%, 50% and, a mixture of 50% ethanol and hydrogenated 10 volumes, D/W 5 min each). Second, 10 drops of Picrosirius were applied and left for 1 h. Third, the blades were washed and dried. Fourth, we applied 10 drops of hematoxilin which was left for 4 min. Finally, the blades were washed and dried once more. Images were taken using optical microscopy (Nykon E200, Tokyo, Japan) and obtained for all samples. The percentage of bone-to-implant contact (BIC%) and the bone area fraction occupancy (BAFO%) inside of the threads were measured using the ImageJ program (National Institute of Health, Bethesda, DC, USA). For the BIC% calculation, the total perimeter around the implant was considered as 100%, and then the areas where the bone was in contact with the implant surface were measured. Whereas for the BAFO% calculation, the total area of threads was measured for the implant model used, and then the percentage of this area of threads occupied by the bone was used.

Statistical analysis: A one-way ANOVA test was used to analyze the statistical differences between the groups. The comparison between the three groups in the same test was performed using the Mann–Whitney U test. These statistical analyses were performed using the software GraphPad Prism 5.01 (GraphPad Software Inc., San Diego, CA, USA). The level of significance was set at α = 0.05.

## 3. Results

### 3.1. In Vitro Characterization of the Surfaces

The SEM images in different increases (500, 1000, 5000 and 10,000×) of the three groups samples analyzed showed microscopic differences in the surface characteristics. However, the EDS (Energy Dispersive Spectroscopy) analysis of all groups showed a surface with high levels of titanium, without identification and/or the presence of other metal ions or contaminants.

**Figure 4:**
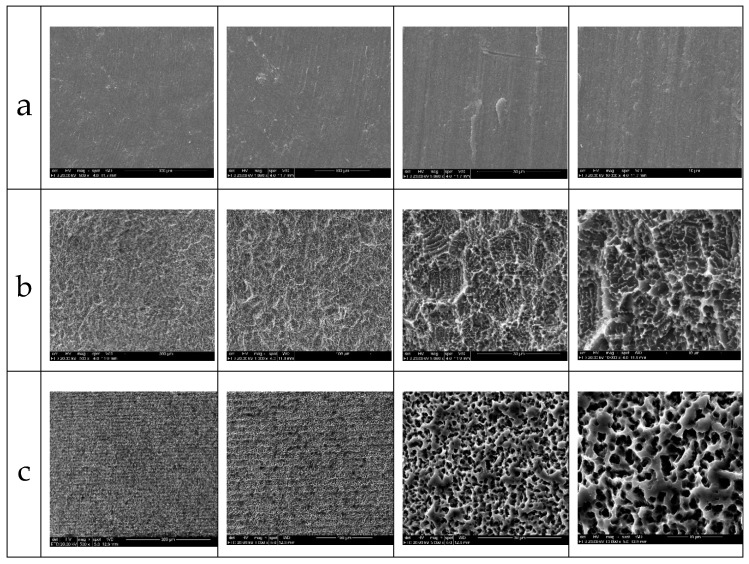
Representative sequence of SEM images of the surface samples of the G1 group (**a**), the G2 group (**b**) and (**c**) the G3 group at different magnification levels (500, 1000, 5000 and 10000×, respectively).

The G1 group (no treated surface) showed a small superficial undulation in the large magnification images produced by the cutting tools. In the G2 group, the surface showed a regular small porosity with a homogeneous distribution. The G3 group showed regularly distributed microgrooves and a similar rugosity to the G2 group samples, due to the same double acid treatment, with a deep and regular morphological pattern with small pores. Representative SEM images of the surface of each group are show in Figure 4.

**Figure 5:**
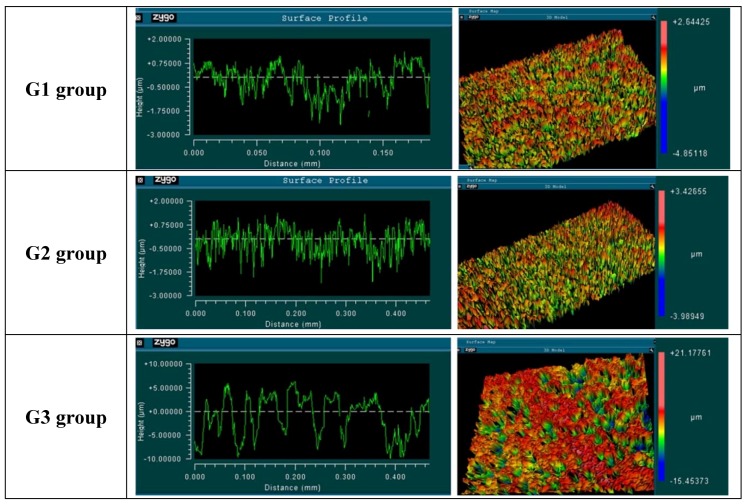
Representative AFM images of the surface sample of G1 group, G2 group and G3 group.

The characteristics of the surface of each group can be observed in the AFM images (Figure 5).

The measured surface roughness showed different values of the analyzed parameters, which are summarized in Table 1.

**Figure 6:**
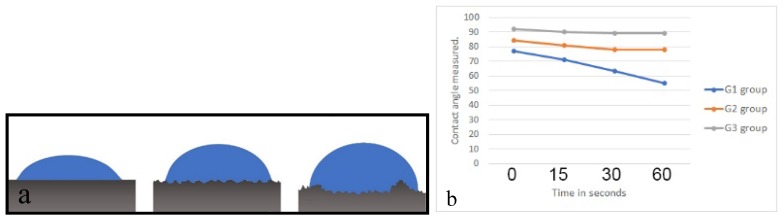
Scheme of the drop behavior on the surface of each group (**a**) and the bar graph of the drop dispersion after the four observation times (**b**).

The wettability analysis revealed the mean value of the contact angle over different periods time, which are presented in the comparative line graph and demonstrated in the schematic image in Figure 6, showing the behavior of the drop on the surface of each group. The G1 group showed a greater wettability of the surface in comparison with the G2 and G3 groups, possibly by the absence of the porosity on the surface.

### 3.2. Histomorphological Analysis and Measurements

After the period determined by the osseointegration (6 weeks), all implant samples presented a good stability, without sample loss or failure of osseointegration. Then, all the implants could be evaluated histologically.

**Figure 7:**
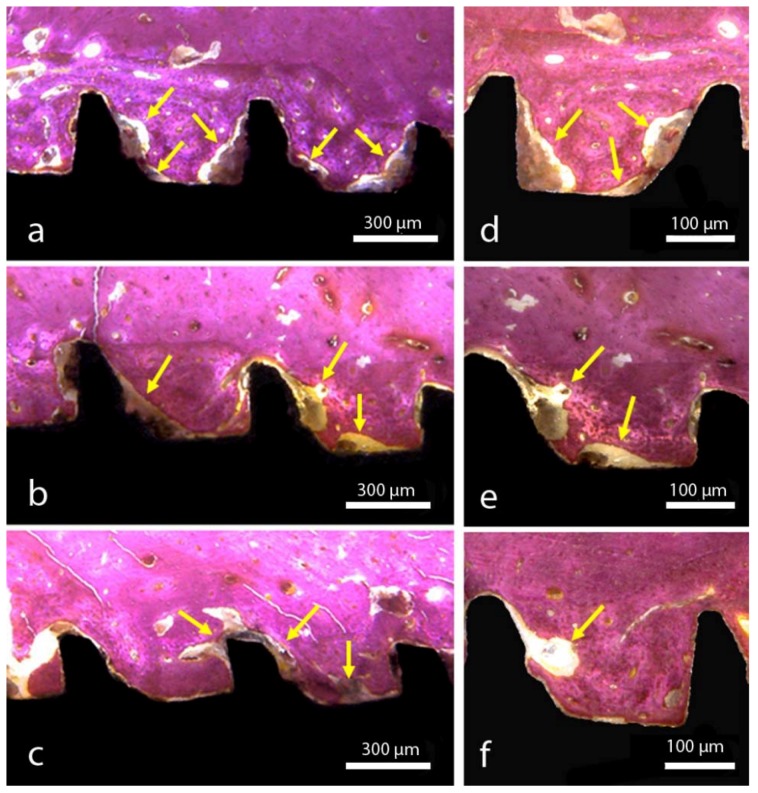
Survey of optical micrograph implant samples placed in different locations. An increase of connective tissue can be observed between (**a**,**d**) group G1, (**b**,**e**) group G2, and (**c**,**f**) group G3. The yellow arrows depict areas with connective tissue.

In the G1 group, a few areas of new bone formation were visible close to the implant surface after the full time period, with poor new bone organization. In the G2 group, a large quantity of new bone formation was observed in comparison with the G1 group, with good organization of new tissue around the implant surface. In the G3 group, a great quantity of new bone formation was observed in comparison with the other two groups, with a good organization and a more advanced bone filling inside the threads in comparison to the other groups. Figure 7 shows a representative image of each group.

Regards to the bone to implant contact (BIC%) and bone area fraction occupancy (BAFO%), the measurement and data analyses are summarized in Table 2.

## 4. Discussion

The treatment to replace fully or partially edentulous patients using titanium implants has been applied with great frequency in dentistry practice, presenting good results and predictability. The production of these implants using titanium as a raw material has shown excellent results from a biological and mechanical point of view. However, the search for new knowledge and the improvement in the behavior of these materials in function, supporting the masticatory loads and the physiological reactions resulting from the medium where they are inserted (buccal medium), continues to challenge science. In this sense, the topographical changes in the surface of the implants have received special attention and are the main area of focus of research in the sense of improving and accelerating the processes of healing of the bone tissue. Thus, the present study had the purpose of comparing differently treated samples, through in vitro and in vivo assays, always using structures with the same macrodesign (disks and implants), but with different surface treatments. Similar with other studies about this subject [1,2,7,14,15,20], the results demonstrated different behavior, both in vitro and in vivo, for the surface treatment models studied, showing that these small changes may modify the host’s biological response.

In the evaluation of the surface of the samples of each group by SEM, different morphological characteristics could be demonstrated, mainly in the G3 group, where the microgrooves were present and distributed in a regular and homogeneous way. As in other studies [21,22,23,24], these microgrooves can be obtained by physical means, such as through the application of a laser, and help to direct cell growth, thus facilitating their organization. However, in the surface model presented here with microgrooves, these microgrooves were produced during the machining of the implants, and consequently, they do not present the possibility of altering the superficial chemical composition, unlike the use of a laser for this purpose [25,26]. The EDS analysis of the samples showed the same surficial composition of all groups tested in the present experiment. The chemical characteristics of the implant surface can directly influence the osteogenic phase that occurs at the interface between the bone tissue and the implant, acting in a number of steps, such as protein adsorption, cell proliferation and differentiation, and bone matrix formation [27,28,29].

The determination of surface roughness parameters of implantable biomaterials samples is an important point for the morphological characterization, considering that this condition will directly influence the adhesion, proliferation, differentiation and other cellular events resulting from the installation of the implant into the bone tissue. Several authors have demonstrated that an adequately machined titanium surface shows a Ra roughness parameter between 0.5 and 1.0 μm [30,31]. However, when the surface receives a treatment by different methods, other values of Ra are to be expected, for example, surfaces treated by acid (Ra variation of 0.54 and 1.97 μm), sandblasted surfaces (Ra variation of 0.84 to 2.12 μm) and in oxidized surfaces (Ra above 2.0 μm). The three different surfaces that were evaluated in this study showed roughness parameter (Ra) values coincidental to the above described values, i.e., for the G1 group, Ra 0.56 ± 0.02 µm, for the G2 group, 0.66 ± 0.05 µm and for the G3 group, 0.67 ± 0.05 µm. However, in G3 group, which presents microgrooves on its surface, the value representing the maximum peak (Rmax) had a much higher value (19.02 ± 3.05 µm) in comparison with the others two groups (7.91 ± 1.48 µm, 6.77 ± 1.63 µm), and consequently, the value of Rz (average of maximum length of peaks and valleys) also had a higher value. These higher peak values (microgrooves) are most likely responsible for the drop behavior in the wettability analysis shown in Figure 6.

**Figure 8:**
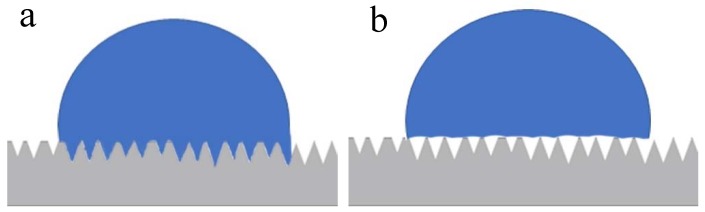
Illustrative scheme of the wettability behavior of a water drop on a rough surface. (**a**) Wenzel’s law of wetting, where the water is in close contact with the surface and (**b**) Cassie’s law of wetting, where air is trapped between parts of the surface and the drop.

Studies on the wettability of surfaces are commonly applied in materials engineering, as there is an influence of the roughness on the wetting properties, which are evaluated by the contact angle measured after the application of fluid on this surface. These studies could be used in many practical applications to adjust surface-fluid interactions [32]. In implantology, the increase of the surface energy and hydrophilicity demonstrates that the examined modifications can accelerate healing between bone tissue and the implant, both in pre-clinical in vivo studies [33,34,35] and in clinical trials [36,37]. In regards to the wettability evaluation of the three groups proposed, the most hydrophilic surface (smaller contact angle) was the G1 group (machined surface), followed by the G2 group, with a porous surface and without microgrooves. The biggest value of the contact angle was presented by the G3 group (with porous and microgrooves in the surface). Interpreting these results, we show in this study that the increase of the rugosity parameters values of Rz and Rmax have a specific influence on the interaction between the water droplet and the surface. To some extent, this makes the droplet behavior on the microgrooves surface different from what is expected on the microscale structured surfaces, based on the validity of either Wenzel’s or Cassie’s law. In this sense, a drop on a rough and hydrophobic surface can adopt two configurations: A Wenzel (complete wetting) and a Cassie configuration (partial wetting) [38,39], as presented in the scheme in Figure 8. In both cases, even if locally, the contact angle does not change (angle of Young) and an increase in the apparent contact angle of the drop is observed. For a superhydrophobic surface, the fundamental difference between the two models is the hysteresis value. For a low roughness, a strong hysteresis value able to reach 100° (Wenzel) is observed and can attributed to an increase in the substrate surface in contact with the drop. It is possible that this characteristic stabilizes faster and more strongly the clot at the surface, consequently favoring the healing of adjacent tissues.

Other studies have shown that surfaces with rugosities have a spreading pattern of slower drop in comparison with less rough surfaces [20,40], with similar results presented in our present study. Regarding the time of observation after the application of the drop on the different surfaces, the present study used a maximum time of 60 s. However, Kulkarni and collaborators [40] observed that the wettability of surfaces with different degrees of roughness for a longer time obtained the same result, that is, the dissipation of the applied drop was inversely proportional to the drop size dropped on the surface. Still, when the drop applied on the surface was observed from a superior view, the G3 group samples show a different orientation of the liquid because of the microgroove lines, where they formed an elliptical form in comparison with the cylindrical form of the G1 and G2 groups.

Studies have shown that the surface topography of an implantable biomaterial can alter the local osteogenic response [41], depending on its roughness, surface energy and chemical characteristics. The osteogenic response can be measured histologically through the evaluation of the bone to implant contact percentage (BIC%) and the bone area fraction occupancy percentage (BAFO%). In this regard, the evaluations of the three titanium implant surfaces after osseointegration in rabbit tibias (6 weeks) showed superior BIC% and BAFO% values for the G3 group, where the principal difference in the surface morphology is the presence of the microgrooves. In this way, Soboyejo et al. [15] presented a study analyzing the cellular behavior on microgrooves, elaborating on the surface of titanium and demonstrating that these grooves guide the adhesion of osteoblastic cells, also inhibiting the growth and migration of fibroblast cells. Moreover, other authors have shown that this type of superficial condition (with microgrooves) can accelerate and increase the growth of bone tissue on the surface of the implant, increasing the retention of the implants [19,21].

## 5. Conclusions

Within the limitations of the present study, the results show that the implants with surfaces modified with microgrooves plus double acid treatment produced a significant enhancement in the process of osseointegration, accelerating healing, increasing the contact between the bone and the implant and the area of new bone formation.

## Figures and Tables

**Table 1:** Mean value and standard deviation (±SD) of the measured roughness and surface parameters in micrometers (µm).

	Parameters	Ra (µm)	Rq (µm)	Rz (µm)	Rmax (µm)
Group					
G1		0.56 ± 0.02	0.75 ± 0.12	5.96 ± 0.42	7.91 ± 0.95
G2		0.66 ± 0.05	0.78 ± 0.10	4.77 ± 0.58	6.77 ± 0.52
G3		0.67 ± 0.05	0.81 ± 0.07	11.02 ± 0.59	19.02 ± 0.88
*p-*value (ANOVA)		0.0081	0.7318	0.0022	0.0045

Ra = Mean roughness. Rq = Quadratic average roughness. Rz = Average peak value of the absolute heights of the 3 highest peaks and the depths of the 3 deepest valleys in terms of roughness. Rmax = Peak maximum of roughness.

**Table 2:** Comparison of measured values of bone to implant contact (BIC%) and bone area fraction occupancy (BAFO%) between the three groups. The data shows the mean, SD, medians and statistical analysis values.

Variables	BIC (%)		BAFO (%)	
Group	Mean ± SD	Median	Mean ± SD	Median
G1	50.45 ± 9.67	50.60	54.87 ± 9.56	54.86
G2	55.32 ± 10.31	55.51	59.09 ± 10.13	59.15
G3	68.65 ± 9.98	68.83	70.12 ± 11.07	70.33
Statistic	*p*-value	--	*p*-value	--
G1 × G2	0.2438	--	0.3078	--
G1 × G3	<0.0001 *	--	0.0005 *	--
G2 × G3	0.0033 *	--	0.0109 *	--

* Statistically significant difference between the group (*p* < 0.05).
